# Deubiquitination of epidermal growth factor receptor by ubiquitin-specific peptidase 54 enhances drug sensitivity to gefitinib in gefitinib-resistant non-small cell lung cancer cells

**DOI:** 10.1371/journal.pone.0320668

**Published:** 2025-04-01

**Authors:** Mi Seong Kim, Min Seuk Kim

**Affiliations:** 1 Department of Oral Physiology, Institute of Biomaterial-Implant, School of Dentistry, Wonkwang University, Iksan, Jeonbuk, Republic of Korea; 2 Wonkwang Dental Research Institute, School of Dentistry, Wonkwang University, Iksan, Jeonbuk, Republic of Korea; National Institute of Cancer Research, TAIWAN

## Abstract

A precise balance between ubiquitination and deubiquitination is crucial for cellular regulation. Ubiquitin-specific peptidase 54 (USP54), an active deubiquitinase (DUB), modulates the ubiquitination of the epidermal growth factor receptor (EGFR). While the significance of USP54 in tumorigenesis is known, its specific function in cancer progression remains unclear. This study investigates the role of USP54 in gefitinib sensitivity in gefitinib-resistant non-small cell lung cancer (NSCLC) cells. Using western blotting and next-generation sequencing, we examined gene expression changes in ubiquitination pathways. USP54 deficiency and its impact on cell viability and gefitinib response were evaluated in 2D and 3D spheroid cancer models. Prolonged gefitinib exposure altered the expression of 20 deubiquitinase-regulating genes. Notably, ubiquitin C-terminal hydrolase L3, downregulated by gefitinib, was identified as a key regulator of EGFR ubiquitination in gefitinib-sensitive PC9 cells. Silencing USP54 in resistant NSCLC cells increased gefitinib-induced EGFR ubiquitination and G0/G1 cell cycle arrest, enhancing drug susceptibility in resistant spheroids. USP54 upregulation in gefitinib-treated cells was associated with reduced EGFR ubiquitination, stabilizing EGFR and promoting cell survival. These findings suggest USP54 as a critical modulator of EGFR stability and a potential therapeutic target to overcome gefitinib resistance in NSCLC.

## Introduction

Abnormalities in the epidermal growth factor receptor (EGFR), such as gene amplification and mutations leading to constant activation, are common in non-small cell lung cancer (NSCLC) [1,2]. EGFR, a member of the erythroblastic oncogene B (ErbB) receptor family with inherent tyrosine kinase activity, is activated by specific ligands, triggering dimerization and phosphorylation, which catalyzes essential signaling pathways for cellular growth and survival [1,2]. After signaling, EGFR is internalized, leading to its degradation and recycling, which ceases its signaling function and facilitates its return to the cell surface [3]. Ubiquitin-mediated endocytosis and degradation of ErbB occur in the absence of ligand binding, especially in cancer cells treated with tyrosine kinase inhibitors (TKIs). Notably, TKIs, such as geldanamycin and erlotinib, facilitate the ubiquitination, endocytosis, and subsequent degradation of ErbB-2 and EGFR [4,5]. Our previous study showed that exposing NSCLC cells to gefitinib triggered EGFR endocytosis and degradation, even in the absence of ligands. This process is regulated by the degree of ubiquitination [6]. Although these studies have highlighted the critical role of TKI-induced EGFR ubiquitination, the intricate molecular mechanisms governing EGFR ubiquitination and deubiquitination in TKI-treated cancer cells remain unclear.

Ubiquitin, a highly conserved protein composed of approximately 76 amino acids, forms covalent bonds with target proteins via an enzymatic cascade involving E1, E2, and E3 enzymes [7]. The balance between ubiquitination and deubiquitination determines the topology and length of the ubiquitin chains, which can widely vary [7]. Dysregulation of this balance has been observed in numerous cancer types [8]. Deubiquitinases (DUBs), which function as counterparts of ubiquitinases, cleave isopeptides and peptide bonds to remove ubiquitin from ubiquitinated proteins [9]. There are approximately 100 DUBs, divided into 7 subgroups, including ubiquitin C-terminal hydrolases (UCHs) and ubiquitin-specific proteases (USPs) [10]. UCH-L3 (UCHL3) is a DUB characterized by a conserved catalytic domain, known as the UCH domain [11]. Its activity has been associated with cancer progression by removing ubiquitin from target substrates, such as the aryl hydrocarbon receptor. UCHL3 expression is upregulated in NSCLC tissues and is associated with poor prognosis in lung adenocarcinoma [12]. USP54, first identified in 2004, was initially categorized as a non-peptidase homolog and presumed to be a catalytically inactive DUB owing to the absence of histidine, which is essential for the catalytic triad in conventional USP domains [13]. However, USP54 upregulation in human colon cancer and its regulatory role in mouse melanoma cell metastasis have highlighted its pro-tumorigenic attributes [14]. Although the significance of USP54 in tumorigenesis has been established, its precise function as a DUB in cancer progression remains unclear. In this study, we aimed to explore the functions of genes involved in DUB activity and their roles in regulating acquired drug resistance mechanisms. Our findings reveal that genes associated with deubiquitination pathways are differentially expressed in response to gefitinib treatment and the development of drug resistance in NSCLC cell lines. Specifically, gefitinib-induced G0/G1 cell cycle arrest and apoptotic cell death in gefitinib-sensitive PC9 cells depend on UCHL3, rather than USP54. In contrast, silencing USP54, which is upregulated during the acquisition of gefitinib resistance, significantly enhances gefitinib efficacy in both 2D cell culture and 3D spheroid models. This study highlights distinct strategies for regulating EGFR ubiquitination based on drug resistance status.

## Materials and methods

### Cell culture, reagents, and transfection

The human NSCLC cell lines PC9 and HCC827, along with their gefitinib-resistant variants (PC9/GR and HCC827/GR), were kindly provided by Dr. Jin Kyung Rho at Asan Medical Center, Ulsan University. All cell lines were cultured in RPMI-1640 medium (HyClone, Logan, UT, USA) supplemented with 10% fetal bovine serum (FBS), 100 U/mL penicillin, and 100 μg/mL streptomycin, and maintained at 37 °C in a 5% CO₂ atmosphere. Gefitinib and antibodies for UCHL3 (#8141s), poly (ADP-ribose) polymerase-1 (PARP; #9542s), ubiquitin (#14049s) and β-actin (#4967s) were obtained from Cell Signaling Technology Inc. (Danvers, MA, USA). The USP54-specific antibody (NBP2-86048) was sourced from Novus Biologicals (Centennial, CO, USA), and EGFR antibodies (sc-373749) were purchased from Santa Cruz Biotechnology (Dallas, TX, USA). The pCMV6 mammalian expression vector containing the full-length UCHL3 gene (pCMV6-UCHL3) was acquired from OriGene Technologies, Inc. (Rockville, MD, USA). For transient gene silencing, synthetic small interfering RNAs targeting UCHL3 (siUCHL3) and USP54 (siUSP54), as well as a scrambled control siRNA, were purchased from Integrated DNA Technologies Inc. (Coralville, IA, USA). The siRNA sequences were as follows: UCHL3, 5′-GGGACAAGAUGUUACAUCAUCAGTA-3′ and 3′-GUCCCUGUUCUACAAUGUAGUAGUCAU-5′; USP54, 5′-CAGUCAAUGGUAAAGGUUAUUCCTT-3′ and 3′-GUGUCAGUUACCAUUUCCAAUAAGGAA-5′; scrambled siRNA, 5′-CGUUAAUCGCGUAUAAUACGCUAT-3′ and 3′-AUACGCGUAUUAUACGCGAUUAACGAC-5′. To validate the knockdown effects, independent siRNAs targeting UCHL3 and USP54 were obtained from separate sources (Bioneer, Daejeon, South Korea). The knockdown efficiency in PC9 and HCC827 cell lines was assessed by Western blot analysis and is shown in S1 Fig. Lipofectamine RNAiMAX and Lipofectamine 3000 (Life Technologies, Carlsbad, CA, USA) were used for siRNA and expression plasmid transfections, respectively, according to the manufacturer’s instructions. Briefly, cells were seeded in 60 mm dishes at a density of 7 ×  10⁵ cells per dish. The following day, expression plasmids (1 μg) and siRNAs (30 nM) were transfected and incubated for 24 and 72 h, respectively. To generate USP54-deficient stable cell lines, sgRNA targeting USP54 (RefSeq NM_152584.6) was designed and synthesized using the pSp-U6-Cas9-2A-Puro plasmid (Macrogen Inc., Seoul, South Korea). Cells were transfected with either control or USP54-specific sgRNA and selected with puromycin (2–10 μg/mL) for 2 weeks. Knockdown was verified by immunoblotting. The sequence for USP54-sgRNA was 5′-GGGGGTCGTGGTAGTGTACAAGG-3′.

### Generation of cancer cell line-derived spheroid

To establish three-dimensional (3D) spheroid cultures of a cancer cell line, cells were resuspended at a density of 6 ×  10^4^ cells/mL in culture medium containing threefold FBS. This suspension was mixed with VitroGel Hydrogel Matrix (Well Bioscience Inc., North Brunswick, NJ, USA) at a 2:1 (v/v) ratio. A 50 μL aliquot of the mixture was dispensed into each well of a U-bottom 96-well plate (S-bio Sumitomo Bakelite Co., Ltd., Houston, NH, USA) and centrifuged at 1,000 ×  *g* for 5 min at 24 °C. After centrifugation, 200 μL of standard culture medium was added to each well, and the plate was incubated at 37 °C in 5% CO₂ for 3 days, allowing the formation of multilayered spheroids for subsequent experiments. Following spheroid formation, cells were incubated with or without gefitinib for an additional 9 days. The culture medium, supplemented with gefitinib or dimethyl sulfoxide (DMSO) as a control, was replaced every 3 days. Images of spheroids were captured using a NIKON Eclipse TS100 microscope (Tokyo, Japan) equipped with a CMOS camera FL-20BW (Tucsen Photonics Co., Ltd., Fujian, China). Image capture was performed using Mosaic 2.4 software (Tucsen Photonics Co., Ltd., Fujian, China), and spheroid parameters, such as diameter, were measured using ImageJ software (version 1.51k, NIH, Bethesda, MD, USA). Spheroid viability was assessed using the Cell Counting Kit-8 (CCK-8; Dojindo Laboratories, Kumamoto, Japan) according to the manufacturer’s instructions. Briefly, 20 μL of CCK-8 solution was added to each well, followed by a 1-h incubation at 37 °C. Absorbance was measured at 450 nm using the iMark microplate reader (Bio-Rad, Hercules, CA, USA). Cell viability was evaluated using the Cyto3D Live-Dead assay Kit (TheWell Bioscience Inc., North Brunswick, NJ, USA). A 2 μL aliquot of Cyto3D reagent was added to 100 μL in each well and incubated at 37 °C for 5–10 min. Fluorescent images were obtained using a LAS A/F microscope (Leica Microsystems, Wetzlar, Germany).

### Next-generation sequencing (QuantSeq 3’ mRNA-seq) analysis

Total RNA was extracted from incubated cells using TRIzol reagent (Invitrogen) and quantified with an ND-2000 spectrophotometer (Thermo Inc., DE, USA). Libraries were prepared using the QuantSeq 3’ mRNA-Seq Library Prep Kit (Lexogen Inc., Austria). Briefly, RNA was hybridized with an oligo-dT primer containing a 5’ Illumina-compatible sequence, followed by reverse transcription and RNA template degradation. Second-strand synthesis was performed using a random primer with a 5’ Illumina-compatible linker. The resulting double-stranded library was purified with magnetic beads to remove residual reaction components, then amplified to include complete adapter sequences for cluster generation. Sequencing was performed in single-end mode with 75 bp reads on the NextSeq 550 system (Illumina, San Diego, CA, USA). QuantSeq 3’ mRNA-Seq reads were aligned using Bowtie2 (Langmead and Salzberg, 2012), with indices generated from genome assembly or transcript sequences to enable genome and transcriptome alignment. The alignment files were used for transcript assembly, abundance estimation, and identification of differentially expressed genes (DEGs). DEGs were identified using coverage data from Bedtools and read counts from unique and multiple alignments [15]. Read count data were normalized with the TMM+CPM method in EdgeR, implemented in the R environment via Bioconductor [16]. Gene classification was performed using the DAVID (http://david.abcc.ncifcrf.gov/) and MEDLINE (http://ncbi.nlm.nih.gov/) databases. Data mining and visualization were conducted with ExDEGA (Ebiogen Inc., Seoul, Korea) and Multi Experiment Viewer (MeV 4.9.0; http://www.tm4.org).

### Immunoprecipitation and Western blotting analysis

Immunoprecipitation (IP) was performed using an IP assay kit (Abcam). Cells were seeded at a density of 2 ×  10^6^ on 100 mm cell culture dishes and incubated under the indicated conditions. Whole cell lysates were prepared using RIPA buffer (Invitrogen) supplemented with proteases and phosphatase inhibitors (Invitrogen), and total protein was quantified using a BCA assay. Cell lysates were incubated with specific antibodies overnight at 4 °C on a rotary mixer. Pre-washed protein A/G Sepharose beads were added to the antibody-coated samples and incubated for 1 h at 4 °C with continuous mixing. The beads were collected by centrifugation at 2,000 ×  g for 2 min at 4 °C and washed three times. Protein complexes were eluted by boiling in 2 × SDS-PAGE loading buffer and analyzed by western blotting. For Western blotting, cell lysates prepared in RIPA buffer were sonicated for 5 sec at 50% power, incubated on ice for 10 min, and centrifuged at 14,440 ×  g for 10 min at 4°C. The supernatant was collected, and protein concentration was determined using BCA assay. Samples (20 μg) were subjected to sodium dodecyl sulfate-polyacrylamide gel electrophoresis (SDS-PAGE) for separation by molecular size. The SDS-PAGE gel concentrations used for each antibody were as follows: 15% for UCHL3; 6% for USP54 and ubiquitin (Ub); and 8% for EGFR, PARP, and β-actin. Electrophoretically separated samples were subsequently transferred onto 0.2-μm PVDF membranes (GE Healthcare Life Sciences, Little Chalfont, UK). The membranes were incubated overnight at 4 °C with primary antibodies against UCHL3, USP54, ubiquitin, EGFR, PARP, and β-actin, each at a 1:1,000 dilution. Afterward, membranes were incubated for 1 h at 23 °C with HRP-conjugated secondary antibodies, including anti-rabbit (sc-2357) and anti-mouse (sc-516102) from Santa Cruz Biotechnology diluted in 1:2000. Immunoreactive proteins were detected using AzureSpot 2.0 software (Azure Biosystems, CA, USA).

### Reverse transcription-quantitative polymerase chain reaction

RNA was extracted from each cell line using TRIzol reagent (Invitrogen, Carlsbad, CA, USA) according to the manufacturer’s instructions. The concentration of total RNA was measured using an ND-2000 spectrophotometer (Thermo Inc., DE, USA). For cDNA synthesis, 1 μg of RNA was reverse-transcribed using the High-Capacity RNA-to-cDNA Kit (Applied Biosystems, Foster City, CA, USA). Quantitative PCR (qPCR) was performed using VeriQuest SYBR Green qPCR Master Mix (Affymetrix, Santa Clara, CA, USA) on an ABI PRISM 7900 Sequence Detection System (Applied Biosystems, Foster City, CA, USA). The following primers were used for qPCR: UCHL3 forward, 5′-TGAATCTGGATCAACCTTGAAAAA-3′, and reverse, 5′- GCTCGTTCTTCAGGGCTCAT-3′; USP54 forward, 5′-TGGGCTGCCTAATGGTGAA-3′, and reverse, 5′-ATATGTCTGGCTCTGCCAACCT-3′; and glyceraldehyde 3-phosphate dehydrogenase (GAPDH) forward, 5′-ATGGAAATCCCATCACCATCTT-3′, and reverse, 5′-CGCCCCACTTGATTT TGG-3′. GAPDH was used as an internal control for normalization to account for variations in mRNA concentration. Relative expression levels were quantified using the 2^-ΔΔCq^ method [17].

### Analysis of cell cycle phases

Flow cytometry was utilized to analyze cell cycle phase distribution. Cells were seeded at a density of 7 ×  10^5^ on 60 mm cell culture dishes. After incubation under the indicated conditions, cells were harvested using 0.05% Trypsin-EDTA (Gibco). Cells were fixed in 70% ethanol at –20 °C for 2 h, then stained with 0.5 mL of propidium iodide/RNase staining buffer (550825; BD Biosciences) for 15 min. At least 2 ×  10^4^ cells were examined using a FACScan analyzer (BD Biosciences).

### Statistical analyses

Statistical evaluation of the data was conducted using Origin 2020 (OriginLab Corporation, Northampton, MA, USA). Results are presented as the means ±  standard deviations, derived from a minimum of three independent experiments. One-way analysis of variance was used to determine statistical significance. For further analysis, Tukey’s post-hoc test was performed. A *p*-value of <  0.05 was considered statistically significant.

## Results

### Prolonged exposure of PC9 cells to gefitinib induces differential expression of genes associated with ubiquitin process

**Fig 1:**
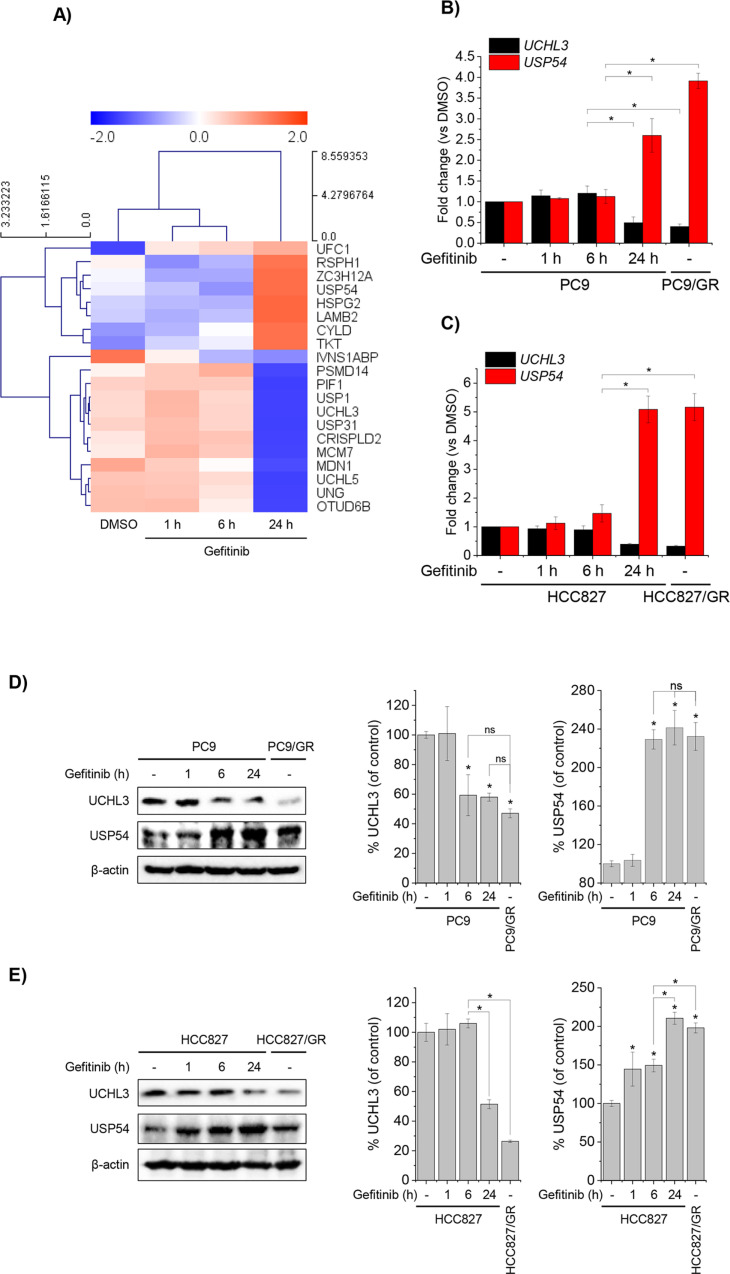
Differential gene expression analysis in NSCLC cells treated with gefitinib.

To investigate how gefitinib affects gene expression in NSCLC cells, QuantSeq 3’ mRNA-Seq analysis was performed on mRNA isolated from PC9 cells treated with gefitinib for 1, 6, and 24 h. Out of 25,737 genes analyzed, 1,846 showed differential expression after 24 h (|fold change|  ≥ 2, p < 0.05). Among these, 20 genes were associated with deubiquitinase (DUB) activity (GO:0101005). Notably, the transcription of eight genes, including CYLD and USP54, increased over time, while others, such as USP1, UCHL3, USP31, USHL5, and OTUD6B, were downregulated compared to controls (DMSO-treated cells) (Fig 1A). Following a detailed next-generation sequencing analysis, we confirmed the effects of gefitinib on UCHL3 and USP54 mRNA and protein levels in NSCLC cell lines. Total RNA and proteins isolated after 1, 6, and 24 h of gefitinib treatment revealed a marked reduction in UCHL3 mRNA levels in PC9 and HCC827 cells after 24 h (Fig 1B and 1C). Conversely, USP54 mRNA levels were significantly elevated. Similar patterns were observed in gefitinib-resistant sublines (PC9/GR and HCC827/GR), with reduced UCHL3 and increased USP54 mRNA levels (Fig 1B and 1C). These mRNA changes were accompanied by corresponding protein level alterations in PC9 and HCC827 cells following prolonged gefitinib treatment (Fig 1D and 1E). These results demonstrate that extended gefitinib exposure induces sustained UCHL3 downregulation and USP54 upregulation in gefitinib-resistant sublines, suggesting a potential interplay between these genes in NSCLC cells under prolonged drug exposure.

Total RNA was extracted from PC9 cells treated with 1 μM gefitinib for 1, 6, and 24 h and analyzed by QuantSeq 3’ mRNA sequencing. (A) Heatmap of differentially expressed genes, clustered hierarchically using Pearson’s correlation (R), with a color scale indicating correlation levels (n =  3). (B, C) mRNA expression levels of UCHL3 and USP54 in PC9 (B) and HCC827 (C) cells, measured by SYBR Green-based RT-qPCR. Data are shown as fold change relative to DMSO-treated controls (*p <  0.05, n =  3). (D, E) Western blot analysis of UCHL3 and USP54 protein levels in PC9 and HCC827 cells. β-Actin served as the loading control. Quantified data on the right are presented as percentages relative to DMSO-treated controls (*p <  0.05, n =  3).

### UCHL3 regulates gefitinib-induced EGFR ubiquitination, degradation, and apoptosis in gefitinib-sensitive PC9 Cells

**Fig 2:**
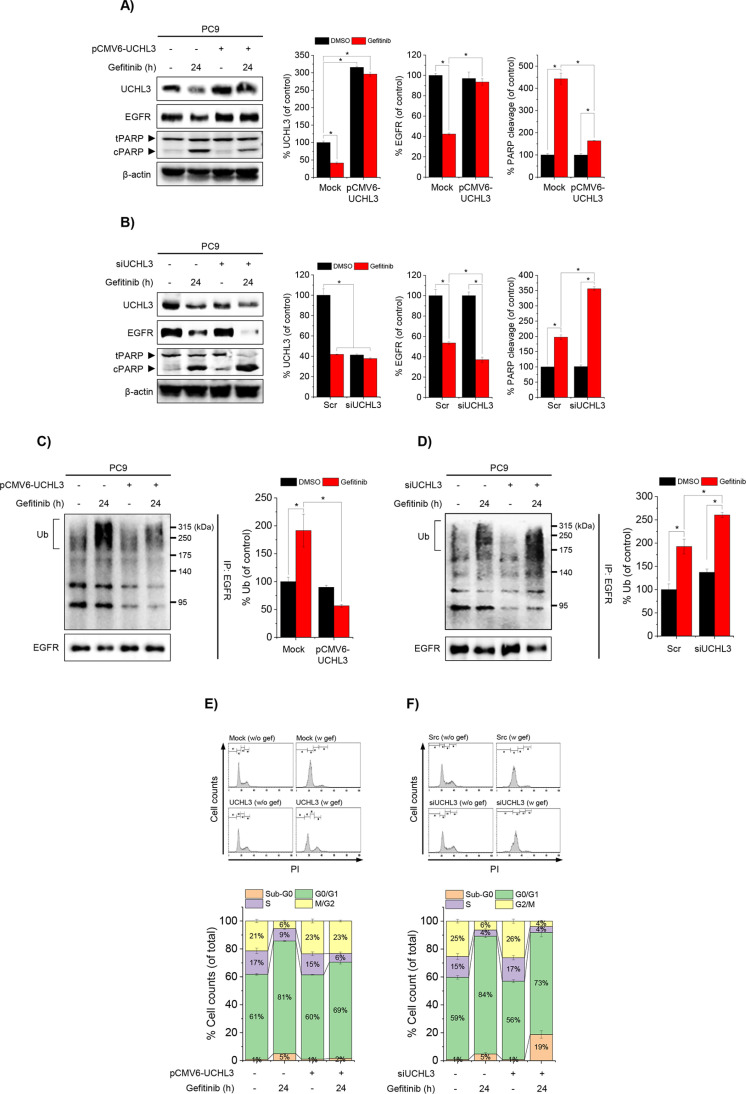
UCHL3 regulates gefitinib-induced EGFR ubiquitination, degradation, and apoptosis in PC9 cells.

To investigate the role of UCHL3 in gefitinib-induced cellular responses, including EGFR ubiquitination, degradation, and cell cycle arrest, PC9 cells were transfected with a UCHL3-encoding expression vector (pCMV6-UCHL3) or siRNA targeting UCHL3 (siUCHL3). Gefitinib-induced downregulation of UCHL3 was reversed by pCMV6-UCHL3 overexpression (Fig 2A). Overexpression of UCHL3 attenuated gefitinib-induced EGFR downregulation and increased PARP cleavage, while UCHL3 knockdown intensified these effects (Fig 2B). We further examined the impact of UCHL3 modulation on EGFR ubiquitination and G0/G1 cell cycle arrest. UCHL3 overexpression reduced gefitinib-induced EGFR ubiquitination, whereas UCHL3 knockdown enhanced it (Fig 2C and 2D). Additionally, UCHL3 overexpression mitigated gefitinib-induced G0/G1 cell cycle arrest in PC9 cells (Fig 2E). While UCHL3 knockdown slightly reduced G0/G1 arrest, it significantly increased the number of cells in the sub-G0 phase, indicative of enhanced apoptotic cell death. These findings underscore UCHL3’s pivotal role in regulating gefitinib-induced EGFR ubiquitination, degradation, and cell cycle dynamics, particularly in gefitinib-sensitive PC9 cells.

PC9 cells were transfected with either pCMV6-UCHL3 or siRNA targeting UCHL3 (siUCHL3) to evaluate the role of UCHL3 in gefitinib-induced processes. (A, B) Effects of UCHL3 overexpression (A) and knockdown (B) on gefitinib-induced EGFR degradation and PARP cleavage. Protein levels of UCHL3, EGFR, and cleaved PARP were quantified relative to DMSO-treated controls (*p <  0.05; n =  3). β-actin served as the loading control (β-actin blot images for 2A and 2B are shown in S2 Fig). (C, D) Impact of UCHL3 modulation on EGFR ubiquitination in gefitinib-treated cells. Whole-cell lysates were immunoprecipitated with anti-EGFR and analyzed by anti-ubiquitin (Ub) blotting. Ubiquitination levels are shown as percentages of DMSO-treated controls (*p <  0.05; n =  3). (E, F) Effects of UCHL3 overexpression (E; n =  4) and knockdown (F; n =  6) on gefitinib-induced cell cycle arrest. Flow cytometry of propidium iodide-stained cells showed the distribution across sub-G0, G0/G1, S, and G2/M phases, expressed as percentages of total cells.

### USP54 deletion enhances gefitinib-induced EGFR ubiquitination and G0/G1 cell cycle arrest in gefitinib-resistant NSCLC cells

**Fig 3:**
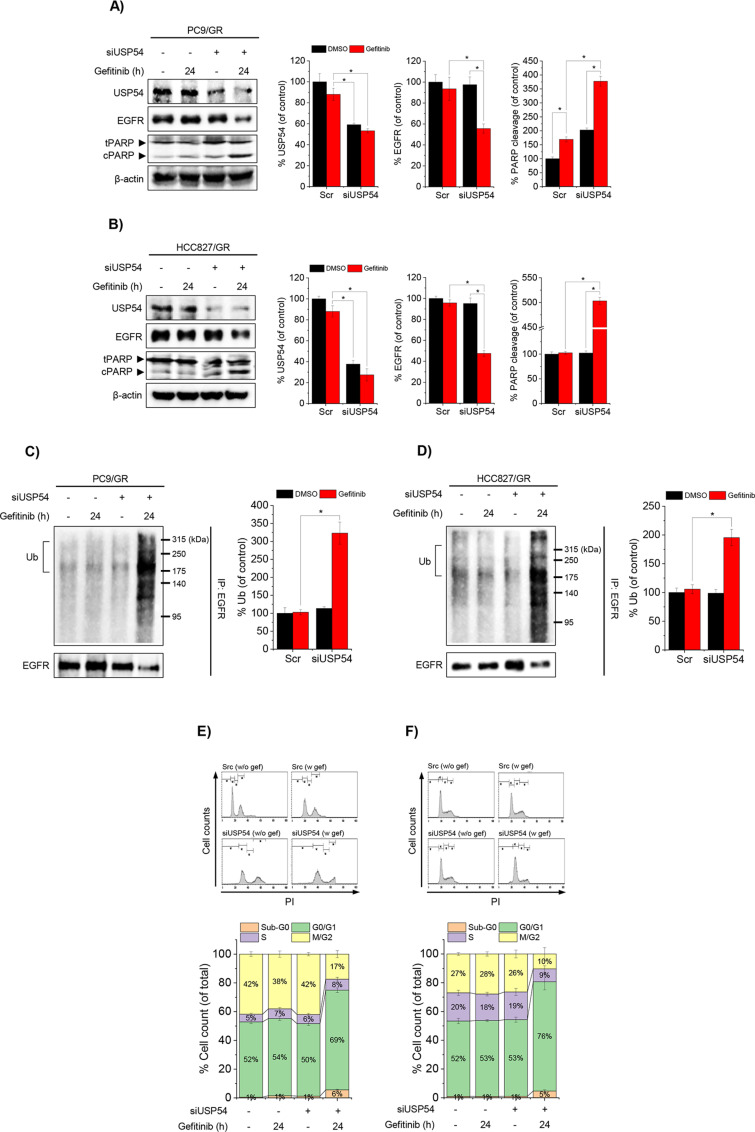
USP54 deficiency enhances gefitinib-induced EGFR ubiquitination, degradation, and apoptosis in gefitinib-resistant NSCLC cells.

The upregulation of USP54 in gefitinib-treated cells, including resistant sublines, suggests its involvement in acquired drug resistance. To evaluate its role, we assessed the effects of USP54 deletion on gefitinib-induced EGFR ubiquitination and cell cycle arrest in the gefitinib-resistant NSCLC cell lines PC9/GR and HCC827/GR. Silencing USP54 with siRNA in PC9/GR and HCC827/GR cells significantly reduced EGFR levels and enhanced PARP cleavage following gefitinib treatment (Fig 3A and 3B). Moreover, USP54 knockdown significantly increased gefitinib-induced EGFR ubiquitination and G0/G1 cell cycle arrest in both cell lines (Fig 3C–3F). These results reveal a compensatory role for USP54 in modulating EGFR ubiquitination and cell cycle regulation, highlighting its contribution to gefitinib resistance in NSCLC cells.

USP54 was silenced using siRNA in gefitinib-resistant PC9/GR and HCC827/GR cell lines to evaluate its role in EGFR regulation and drug sensitivity. (A, B) Effects of USP54 knockdown on gefitinib-induced EGFR degradation and PARP cleavage in PC9/GR (A) and HCC827/GR (B) cells. Protein levels of USP54, EGFR, and cleaved PARP were quantified relative to DMSO-treated controls (*p <  0.05; n =  3). β-Actin served as the loading control (β-actin blot images for 3A and 3B are shown in S3 Fig). (C, D) Impact of USP54 deficiency on EGFR ubiquitination in PC9/GR (C) and HCC827/GR (D) cells following gefitinib treatment. Whole-cell lysates were immunoprecipitated with anti-EGFR and analyzed by anti-ubiquitin (Ub) blotting, with results expressed as percentages relative to controls (*p <  0.05; n =  3). (E, F) Changes in cell cycle arrest induced by gefitinib in USP54-deficient PC9/GR (E; n =  4) and HCC827/GR (F; n =  4) cells. Flow cytometry of propidium iodide-stained cells showed distributions across cell cycle phases, expressed as percentages of total cells (*p <  0.05).

### USP54 deficiency enhances gefitinib sensitivity in 3D spheroids derived from PC9/GR cells

**Fig 4:**
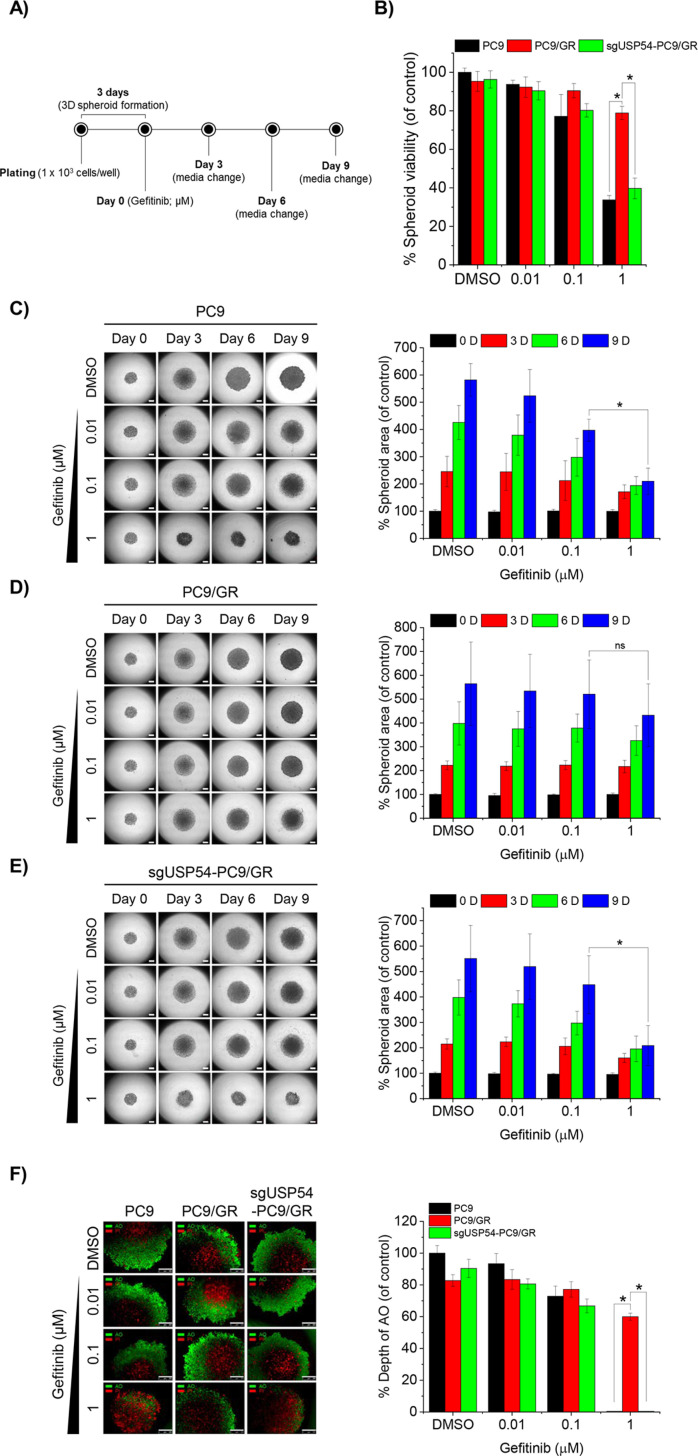
USP54 deficiency reduces spheroid viability, size, and proliferative zone in gefitinib-treated PC9/GR cells.

To evaluate the impact of USP54 deficiency on gefitinib resistance in three-dimensional (3D) systems, spheroids were generated from PC9 and PC9/GR cell lines. PC9/GR cells were transfected with sgRNA targeting USP54 (sgUSP54), and spheroids were formed in a hydrogel matrix over 3 days. Gefitinib was then applied at 1, 3, and 10 μM concentrations, and spheroid proliferation and viability were assessed using CCK-8 and Cyto3D staining on day 9 (Fig 4A). PC9 spheroids exhibited significant sensitivity to 1 μM gefitinib, with viability reduced to 34% compared to DMSO-treated controls. In contrast, PC9/GR spheroids showed marked tolerance to gefitinib. However, USP54 deletion in PC9/GR spheroids restored gefitinib sensitivity, reducing viability to levels comparable to PC9 spheroids (Fig 4B). Over 9 days, gefitinib significantly reduced the overall size and depth of proliferative zones in PC9 spheroids, whereas PC9/GR spheroids were less affected. Deletion of USP54 in PC9/GR spheroids reversed this resistance, resulting in gefitinib-induced reductions in spheroid size and proliferative depth comparable to those seen in PC9 spheroids (Fig 4C–4F). These findings demonstrate that USP54 deficiency restores gefitinib sensitivity in resistant PC9/GR spheroids, reducing their viability and proliferation to levels observed in nonresistant spheroids.

(A) Schematic of spheroid formation and gefitinib treatment. PC9, PC9/GR, and sgUSP54-PC9/GR cells were embedded in a hydrogel matrix and plated in ultra-low attachment U-shaped 96-well plates (1 ×  10³ cells/well). Spheroids formed over 3 days were treated with gefitinib at the indicated concentrations every 3 days. (B) Spheroid viability on day 9 post-gefitinib treatment was assessed using the Cell Counting Kit-8 (CCK-8) assay, with results shown relative to DMSO-treated Scr-PC9 controls (*p <  0.05, n =  3). (C–E) Brightfield images of spheroids derived from PC9 (C), PC9/GR (D), and sgUSP54-PC9/GR (E) cells were captured every 3 days. Spheroid areas were measured and expressed as percentages of DMSO-treated controls on day 0 (*p <  0.05, n =  3). Scale bar =  400 μm. (F) Fluorescent images of spheroids after 9 days of gefitinib treatment were obtained using Acridine orange (live cells) and propidium iodide (dead cells) staining. The depth of the proliferative zone was measured and shown as percentages of DMSO-treated PC9 controls (*p <  0.05, n =  3). Scale bar =  500 μm.

## Discussion

Evidence, including findings from this study, demonstrates that anticancer drugs like TKIs not only block EGFR phosphorylation and downstream signaling but also promote EGFR degradation by enhancing its ubiquitination, thereby reducing proliferative signals [4,18]. This dual role of TKIs highlights deubiquitinases (DUBs) as potential therapeutic targets for modulating cancer cell sensitivity to TKIs. Numerous studies have shown that pharmacological inhibition of DUBs enhances TKI sensitivity and offers a promising strategy to overcome drug resistance [19–21]. While DUBs play a critical role in acquired resistance, the specific DUBs regulating deubiquitination in TKI-resistant cancers remain largely unidentified. In this study, we demonstrated that prolonged gefitinib exposure in NSCLC cells induces differential regulation of genes, including those related to DUB activity. Notably, the upregulation of USP54 reduced gefitinib-induced EGFR ubiquitination in resistant cells. Our results revealed that UCHL3 and USP54, two DUB-encoding genes, are oppositely regulated in response to gefitinib (Fig 1D, 1E). Gefitinib-induced ubiquitination in sensitive cells was predominantly associated with UCHL3 (Fig 2C–2F), whereas in resistant cells, USP54 played a compensatory role in reducing EGFR ubiquitination (Fig 3). Suppressing USP54 in gefitinib-resistant cells reversed gefitinib insensitivity, restoring EGFR ubiquitination and reducing resistance (Figs 3 and 4). These findings highlight a complex interplay between USP54 and EGFR degradation pathways, suggesting that USP54 sustains EGFR signaling under gefitinib-induced stress and contributes to drug resistance mechanisms.

USP54, a non-protease homolog of the USP family due to its lack of a catalytic histidine residue, raises intriguing questions about its role in EGFR deubiquitination. Although USP54 is dispensable for normal mouse survival, as indicated by knockout models, its upregulation in chemically induced colon carcinoma promotes invasion and metastasis [14]. This suggests that USP54, while nonessential in normal physiology, plays a significant role in cancer progression and therapy resistance. Consistently, we observed that USP54 deletion in gefitinib-resistant NSCLC cells enhanced EGFR ubiquitination in response to gefitinib (Fig 3C and 3D). These findings underscore USP54’s function as a selective DUB, likely capable of differentiating substrates based on the context of cancer progression. Studies involving USP54-deficient mouse models have also shown a lower incidence of invasive adenocarcinoma but a higher incidence of dysplasia, further emphasizing USP54’s complex role in cancer [14].

Our study also revealed that USP54 deficiency increases gefitinib sensitivity in both 2D cell cultures and 3D spheroid systems. 3D spheroids, which better replicate *in vivo* conditions compared to 2D systems, provide a more physiologically relevant model for studying drug resistance and testing therapeutics [22,23]. The outer proliferative layers and inner hypoxic zones of 3D spheroids mimic solid tumor characteristics, making them valuable tools for identifying mechanisms of drug resistance [23]. Although not explored in detail here, the observed enhanced sensitivity to gefitinib upon USP54 knockdown suggests that targeting USP54 alongside EGFR may offer a promising dual-inhibition strategy to overcome NSCLC resistance.

## Conclusions

In conclusion, the significance of this study lies in its potential implications for understanding the mechanisms underlying acquired resistance to EGFR-TKIs in NSCLC. Our data suggest that differential deubiquitination pathways involving UCHL3 and USP54 are potential targets for therapeutic interventions. Future studies are required to validate these findings in clinical samples and explore the feasibility of targeting USP54 to overcome gefitinib resistance in NSCLC. Our study provides valuable insights into the molecular mechanisms underlying drug resistance in NSCLC and proposes new avenues for therapeutic intervention. Manipulating DUB activity, specifically through USP54, offers a promising strategy for counteracting gefitinib resistance, potentially leading to improved clinical outcomes in patients with resistant forms of NSCLC.

## Supporting information

S1 Fig Validation of knockdown efficiency of siRNAs targeting UCHL3 and USP54 from independent sources. Independent siRNAs targeting UCHL3 (siUCHL3) and USP54 (siUSP54) were purchased from Bioneer (Daejon, South Korea). The siRNA sequences were as follows: siUCHL3 #1, 5′-GAGGAAUCUGUGUCAAUGA-3′ and 3′-UCAUUGACACAGAUUCCUC-5′ and siUCHL3 #2, 5′-CCUGUGGAACAAUUGGAU-3′ and 3′-AGUCCAAUUGUUCCACAGG-5′ and siUCHL3 #3, 5′-CCUCUUUUCUUGUGAAGGA-3′ and 3′-UCCUUCACAAGAAAAGAGG-5′; siUSP54 #1, 5′-CUGAGAGCUCAAAUGUCUA-3′ and 3′-UAGACAUUUGAGCUCUCAG-5′ and siUSP54 #2, 5′-GAGACAGUCAGCAAUAUGA-3′ and 3′-UCAUAUUGCUGACUGUCUC-5′ and siUSP54 #3, 5′-CACUUCCACAUUGCUGAUG-3′ and 3′-CAUCAGCAAUGUGGAAGUG-5′. (A-D) Each siRNA targeting UCHL3 and USP54 was transfected into PC9 and HCC827 cells and incubated for 72 h. Whole cell lysates were collected and analyzed for endogenous UCHL3 and USP54 levels. β-actin served as a loading control.
(TIF)

S2 Fig . β-actin blot images for UCHL3 and EGFR. PC9 cells were transfected with either pCMV-UCHL3 or siRNA targeting UCHL3 (siUCHL3). After incubation with or without gefitinib for 24 h, whole cell lysates were collected and analyzed by Western blot. (A-B) β-actin primary antibody (1:1000) was used as a loading control for UCHL3 and EGFR shown in Fig 2A and 2B.
(TIF)

S3 Fig β-actin blot images for USP54 and EGFR. PC9/GR and HCC827/GR cells were transfected with siRNA targeting USP54 (siUSP54). After incubation with or without gefitinib for 24 h, whole cell lysates were collected and analyzed by Western blot. (A-B) β-actin primary antibody (1:1000) was used as a loading control for USP54 and EGFR shown in Fig 3A and 3B.
(TIF)

S1 File QuantSeq 3’ mRNA-sequencing. (XLSX)

S2 File All raw data used for quantification. (ZIP)

S1 Raw Images Uncropped original blot images for all Western blots. (PDF)
