# Supplementary figures and images for: Deubiquitination of epidermal growth factor receptor by ubiquitin-specific peptidase 54 enhances drug sensitivity to gefitinib in gefitinib-resistant non-small cell lung cancer cells
Source: PLoS One. 2025 Apr 1;20(4):e0320668. doi: 10.1371/journal.pone.0320668 (PMC11960930; doi:10.1371/journal.pone.0320668)

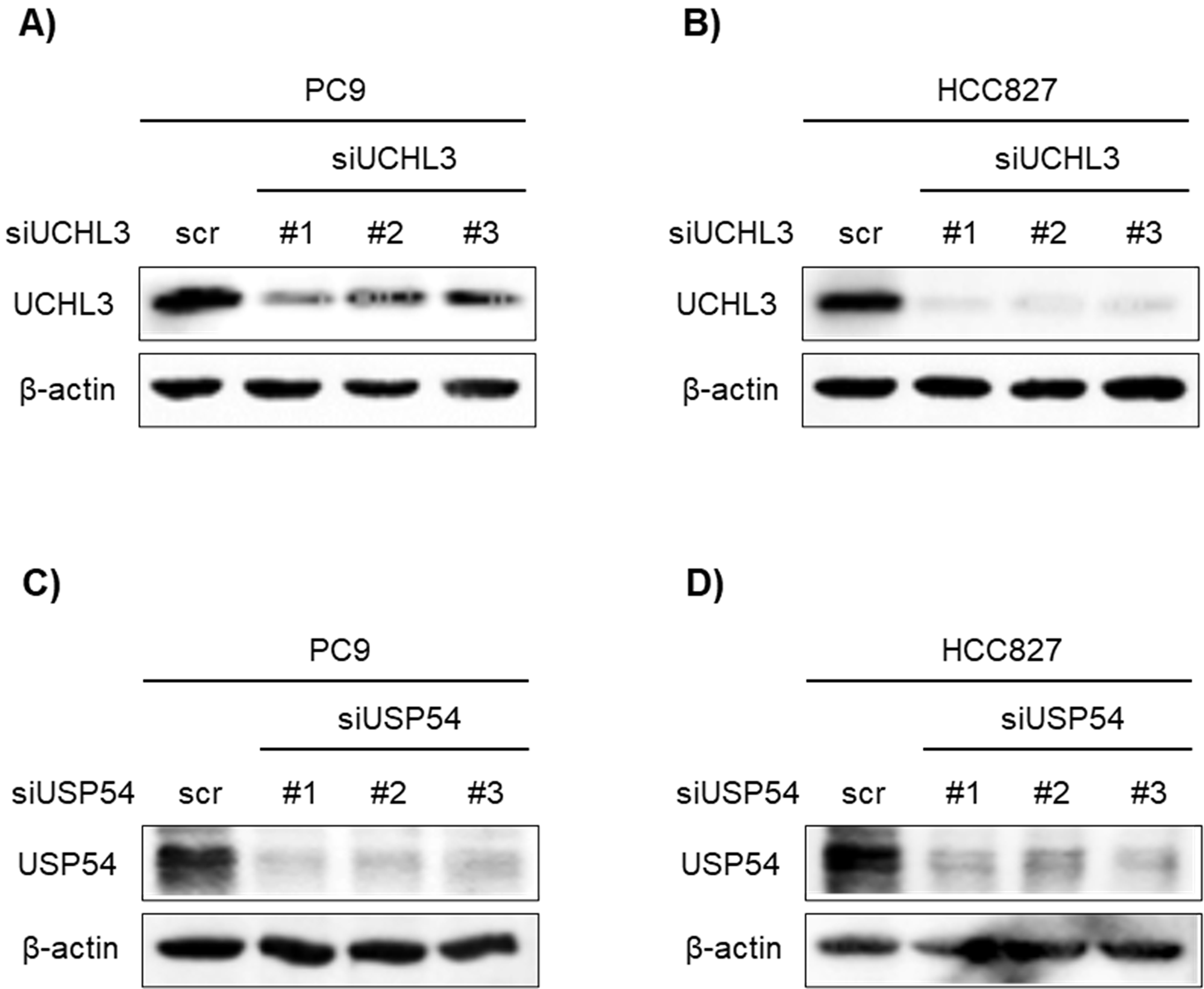

Supplement: S1 Fig — Independent siRNAs targeting UCHL3 (siUCHL3) and USP54 (siUSP54) were purchased from Bioneer (Daejon, South Korea). The siRNA sequences were as follows: siUCHL3 #1, 5′-GAGGAAUCUGUGUCAAUGA-3′ and 3′-UCAUUGACACAGAUUCCUC-5′ and siUCHL3 #2, 5′-CCUGUGGAACAAUUGGAU-3′ and 3′-AGUCCAAUUGUUCCACAGG-5′ and siUCHL3 #3, 5′-CCUCUUUUCUUGUGAAGGA-3′ and 3′-UCCUUCACAAGAAAAGAGG-5′; siUSP54 #1, 5′-CUGAGAGCUCAAAUGUCUA-3′ and 3′-UAGACAUUUGAGCUCUCAG-5′ and siUSP54 #2, 5′-GAGACAGUCAGCAAUAUGA-3′ and 3′-UCAUAUUGCUGACUGUCUC-5′ and siUSP54 #3, 5′-CACUUCCACAUUGCUGAUG-3′ and 3′-CAUCAGCAAUGUGGAAGUG-5′. (A-D) Each siRNA targeting UCHL3 and USP54 was transfected into PC9 and HCC827 cells and incubated for 72 h. Whole cell lysates were collected and analyzed for endogenous UCHL3 and USP54 levels. β-actin served as a loading control. (TIF) [file pone.0320668.s001.tif]

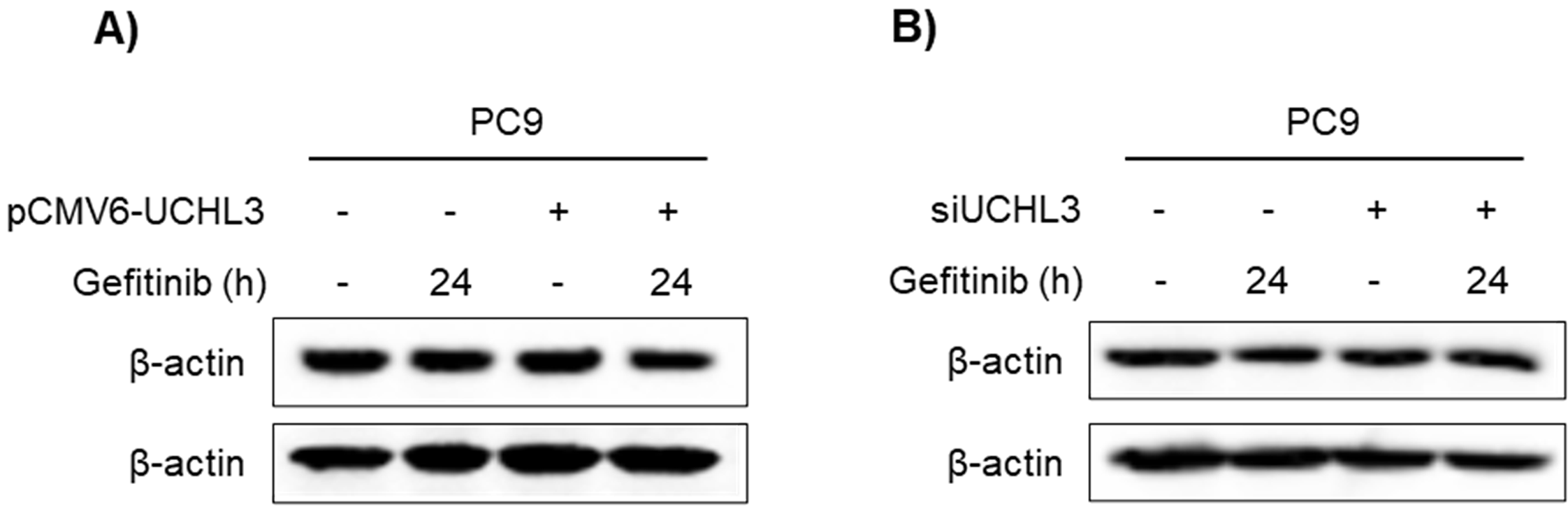

Supplement: S2 Fig — PC9 cells were transfected with either pCMV-UCHL3 or siRNA targeting UCHL3 (siUCHL3). After incubation with or without gefitinib for 24 h, whole cell lysates were collected and analyzed by Western blot. (A-B) β-actin primary antibody (1:1000) was used as a loading control for UCHL3 and EGFR shown in Fig 2A and 2B. (TIF) [file pone.0320668.s002.tif]

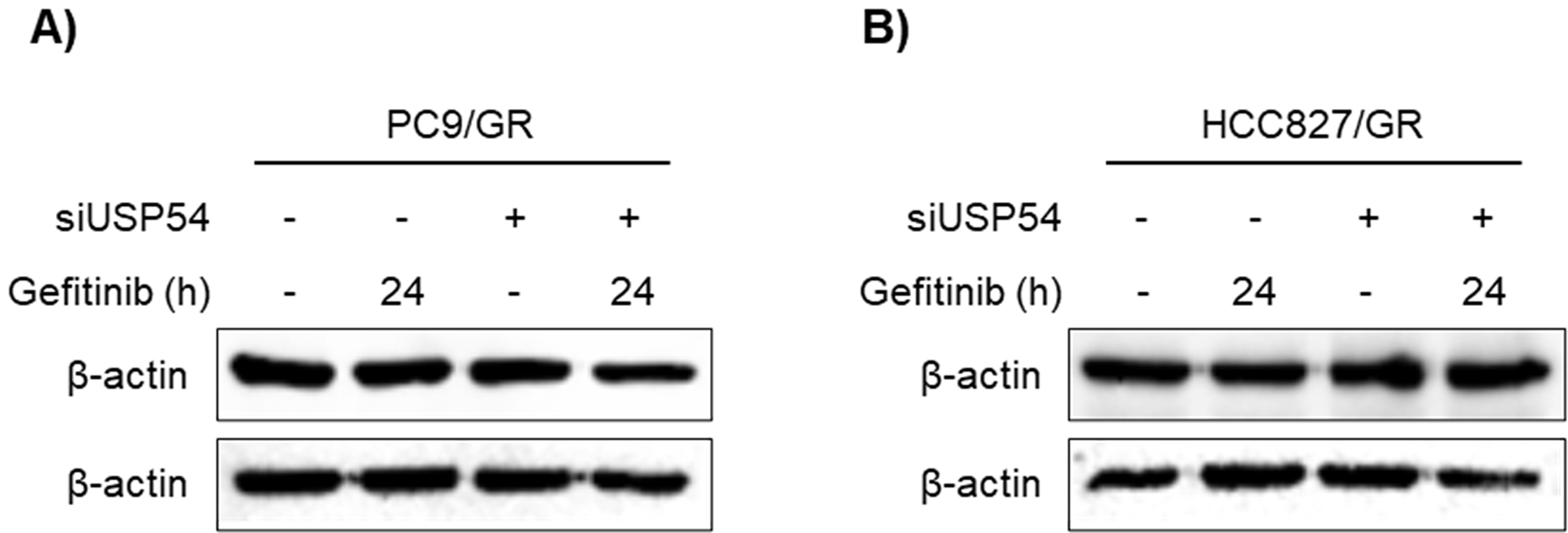

Supplement: S3 Fig — PC9/GR and HCC827/GR cells were transfected with siRNA targeting USP54 (siUSP54). After incubation with or without gefitinib for 24 h, whole cell lysates were collected and analyzed by Western blot. (A-B) β-actin primary antibody (1:1000) was used as a loading control for USP54 and EGFR shown in Fig 3A and 3B. (TIF) [file pone.0320668.s003.tif]

Figure 1

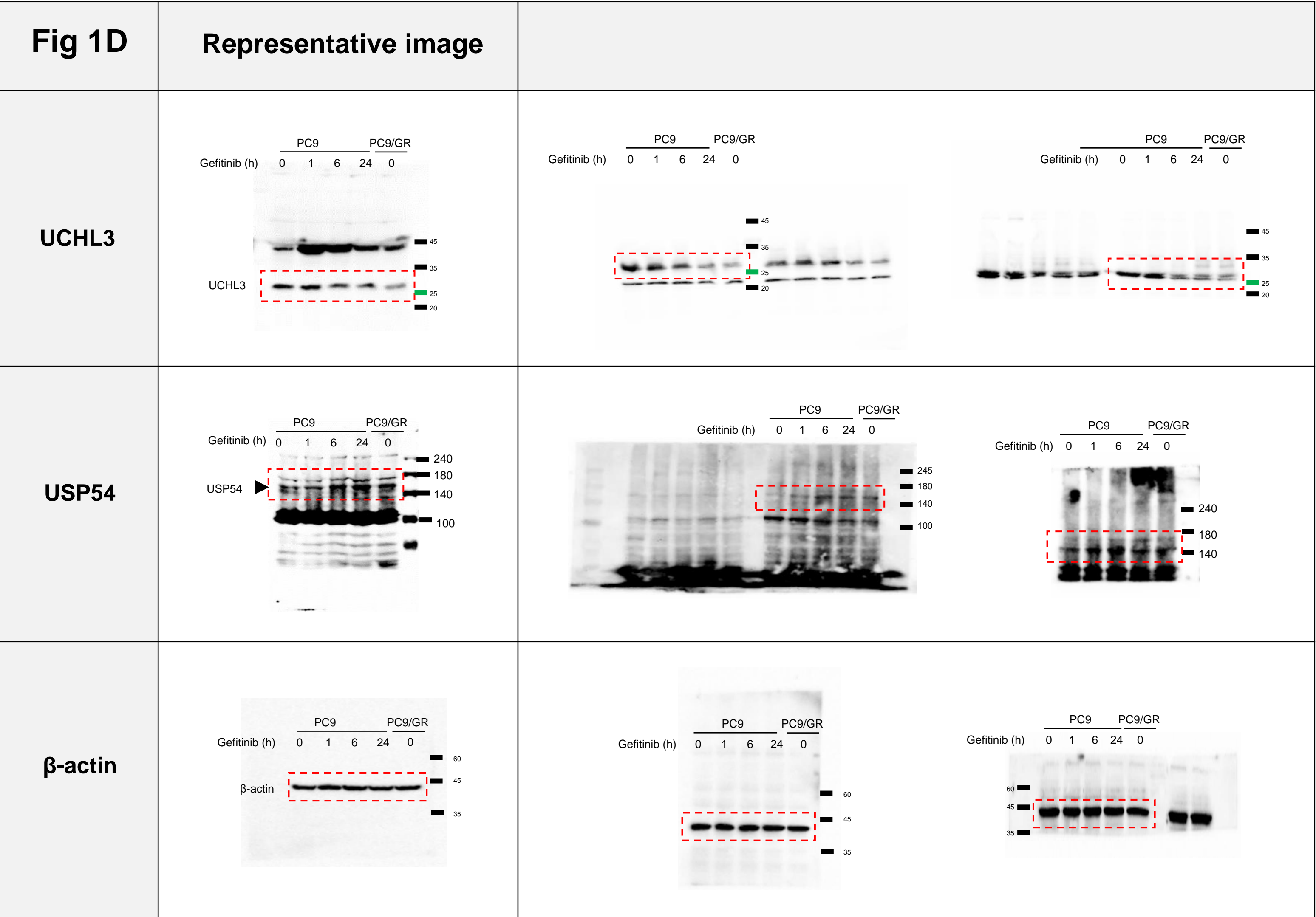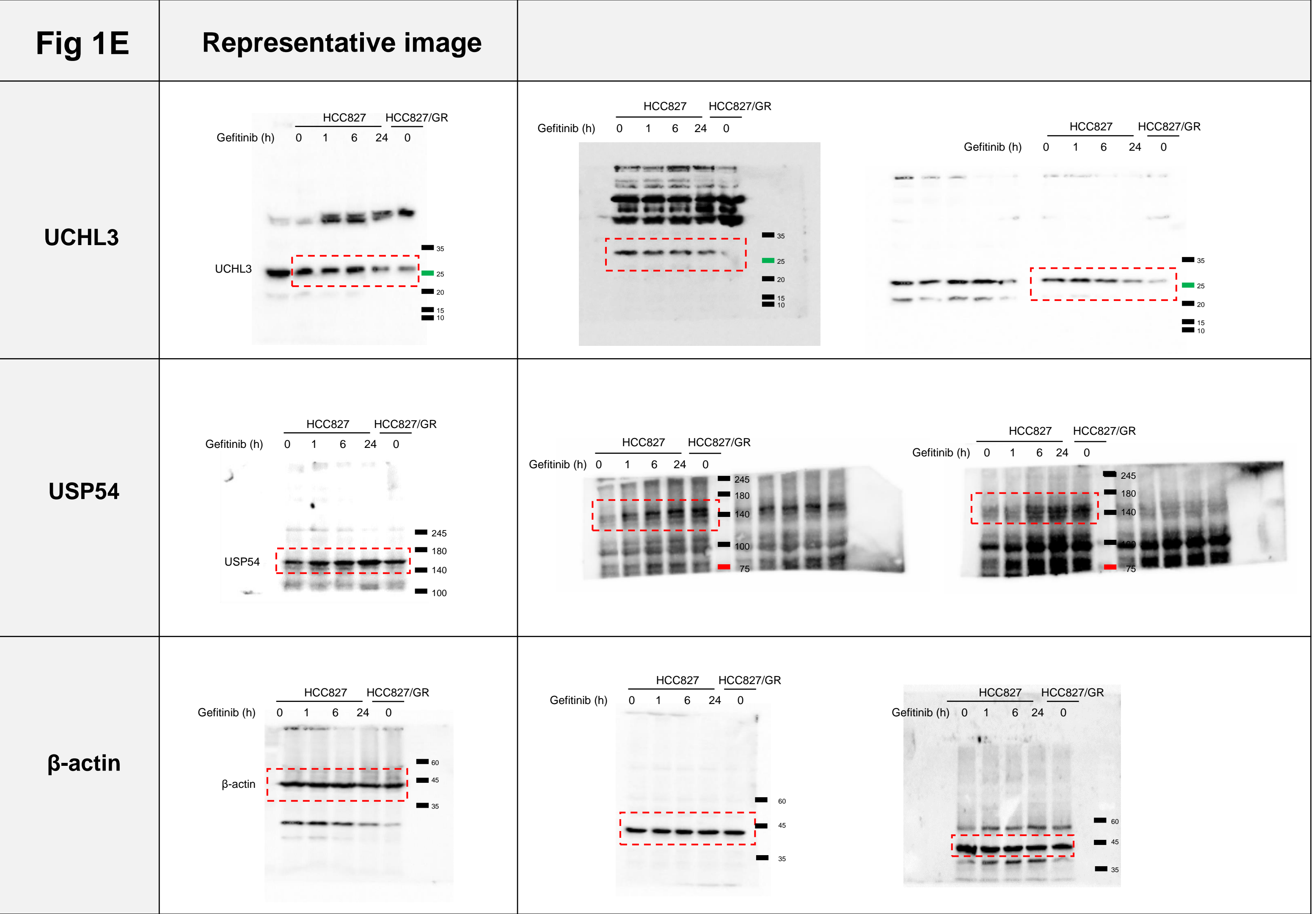

Figure 2

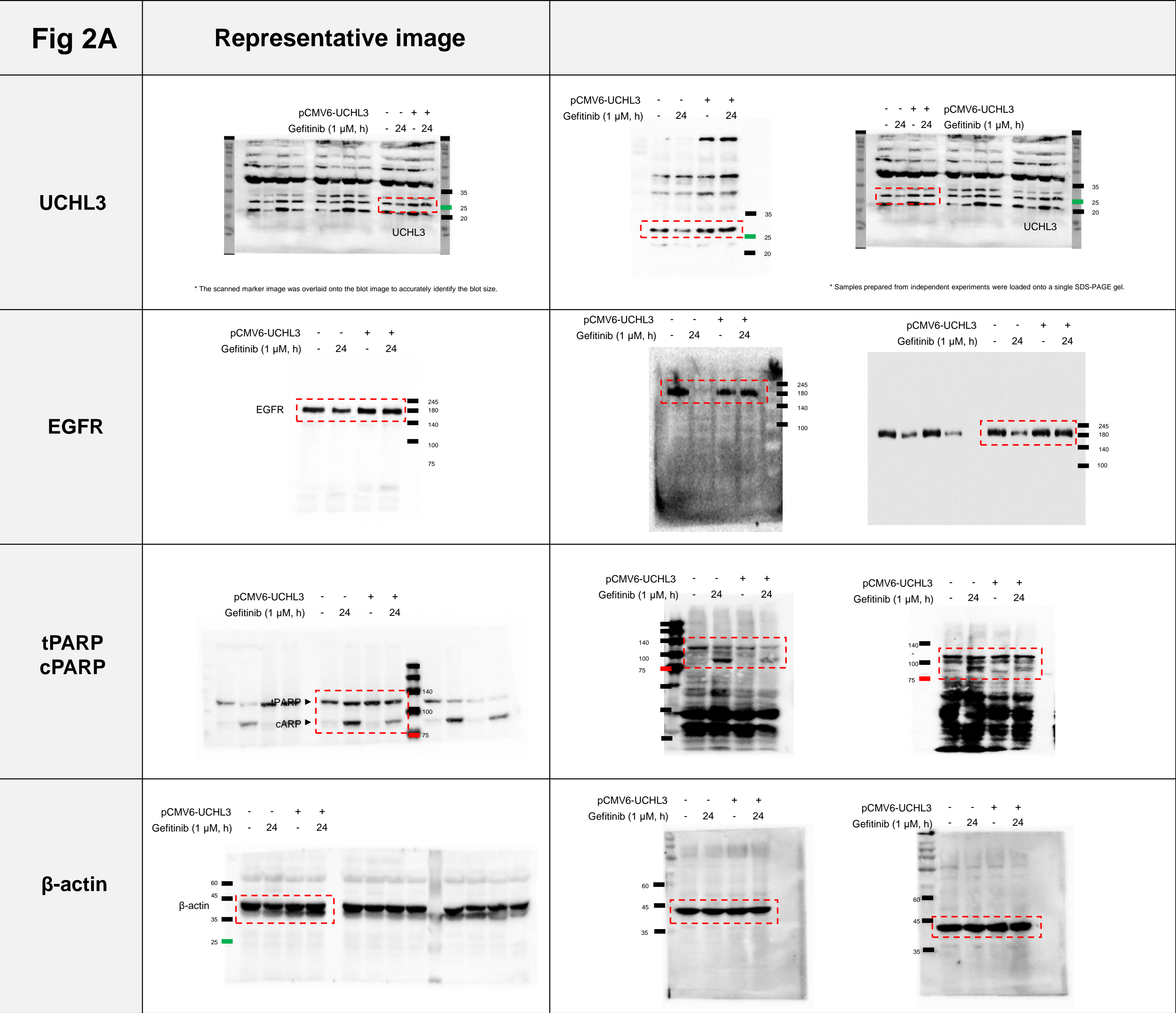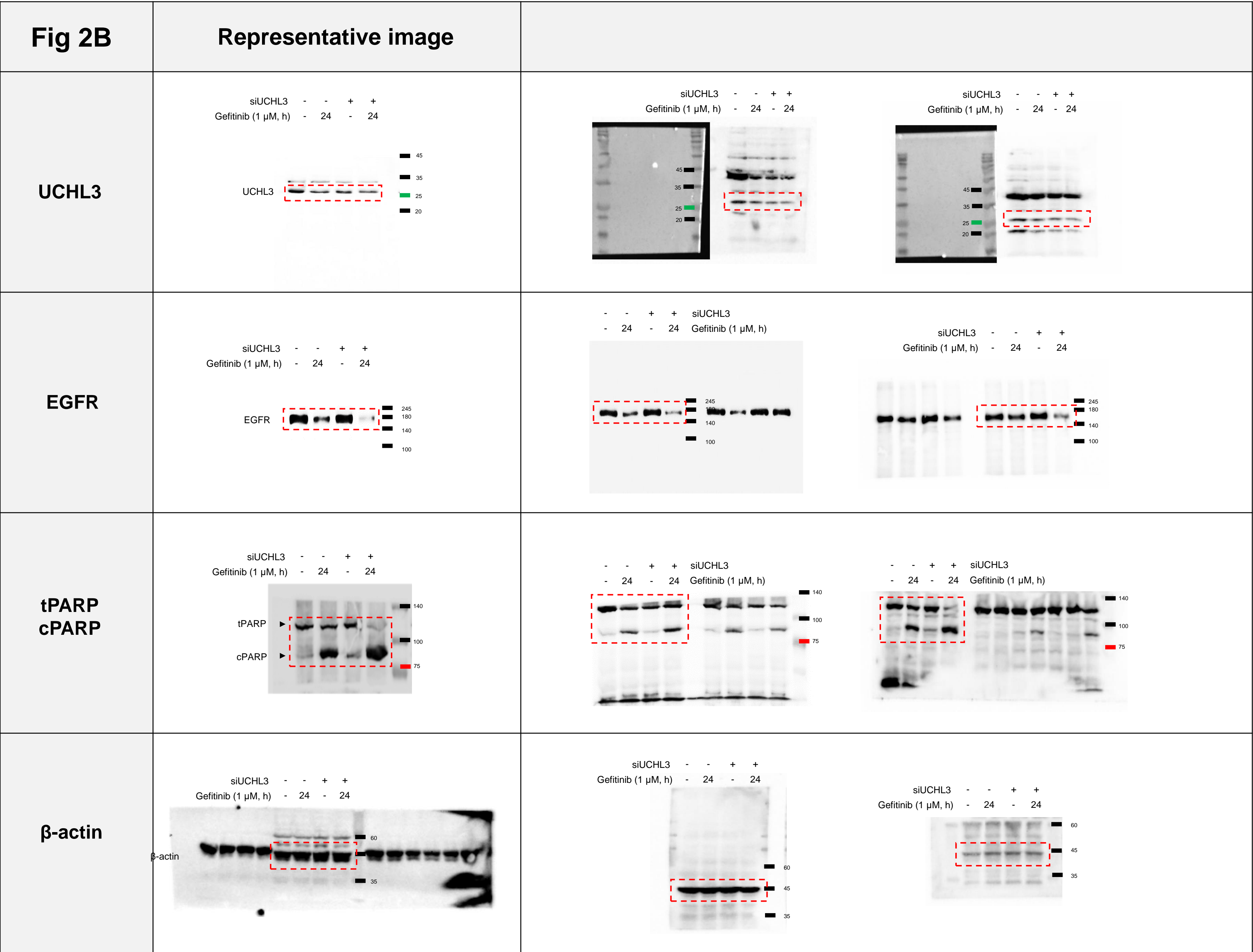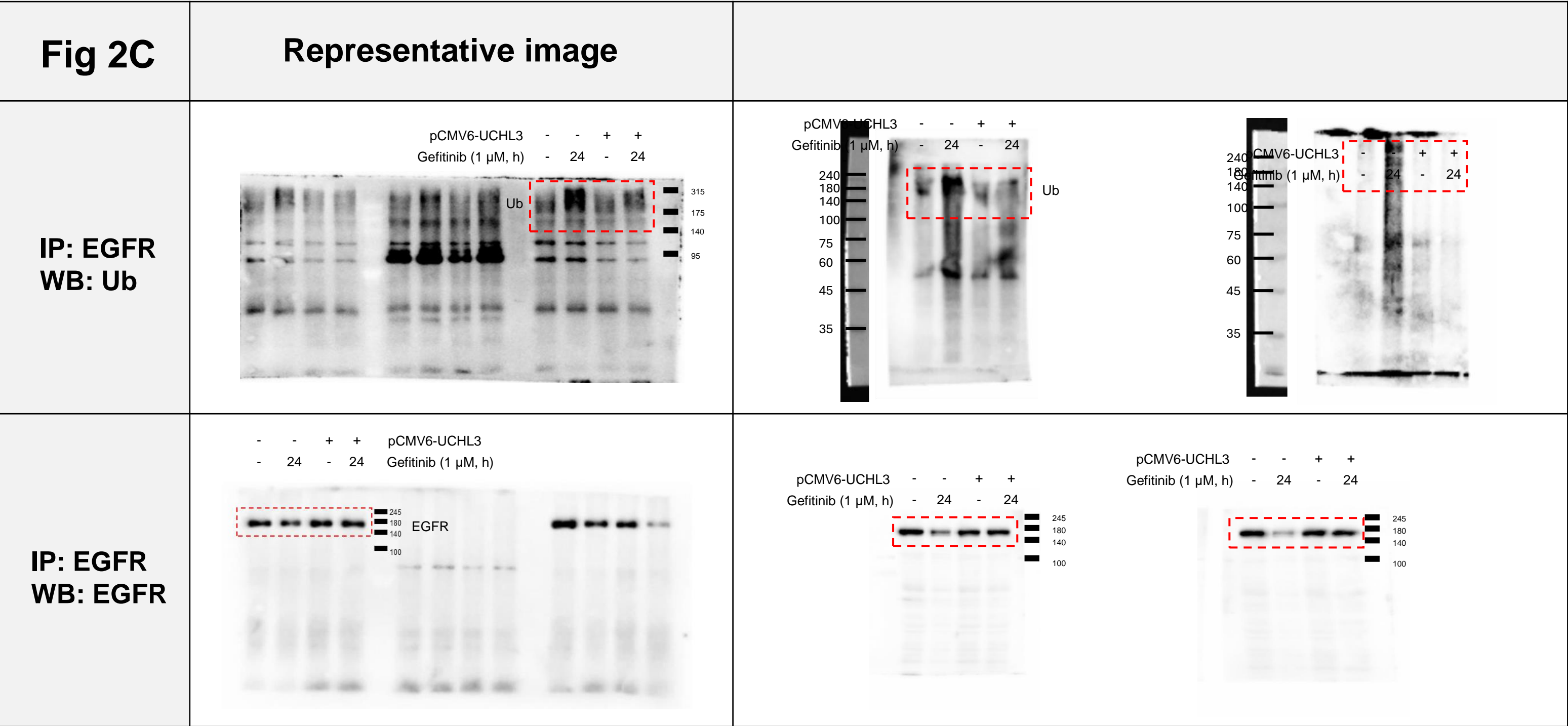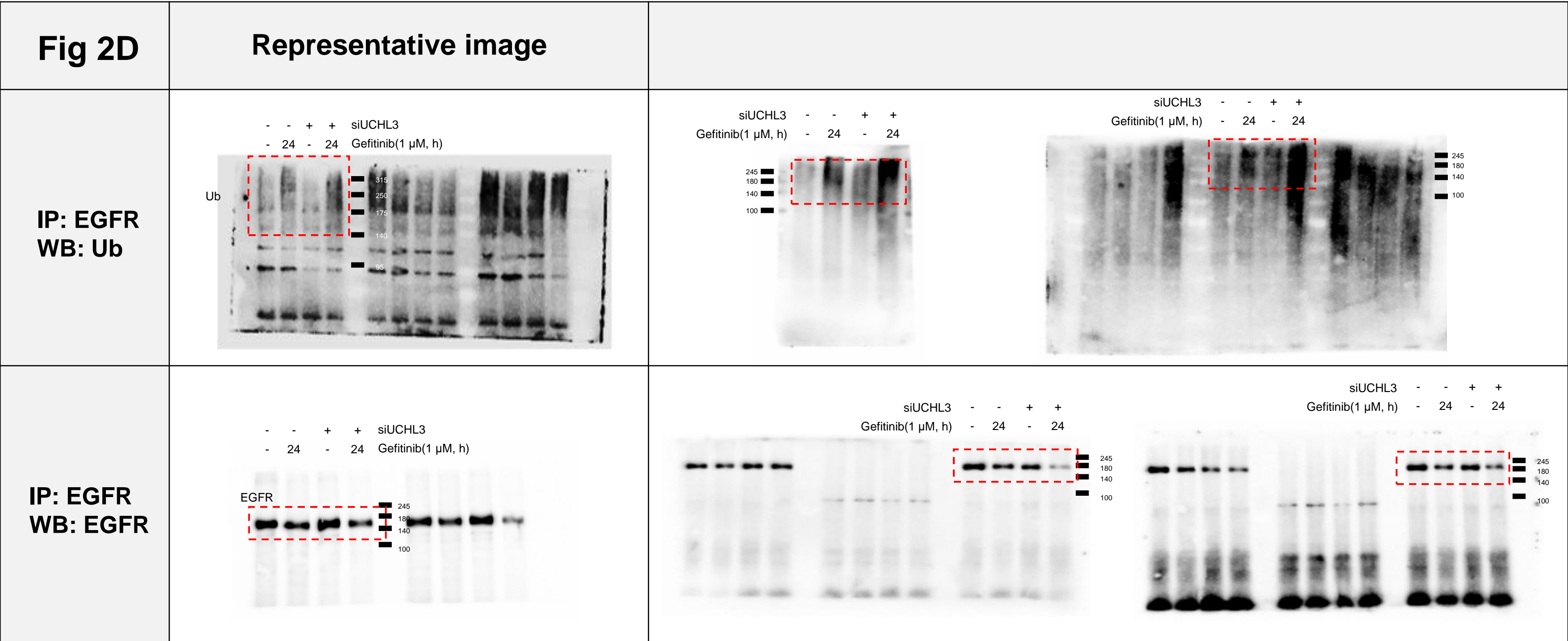

Figure 3

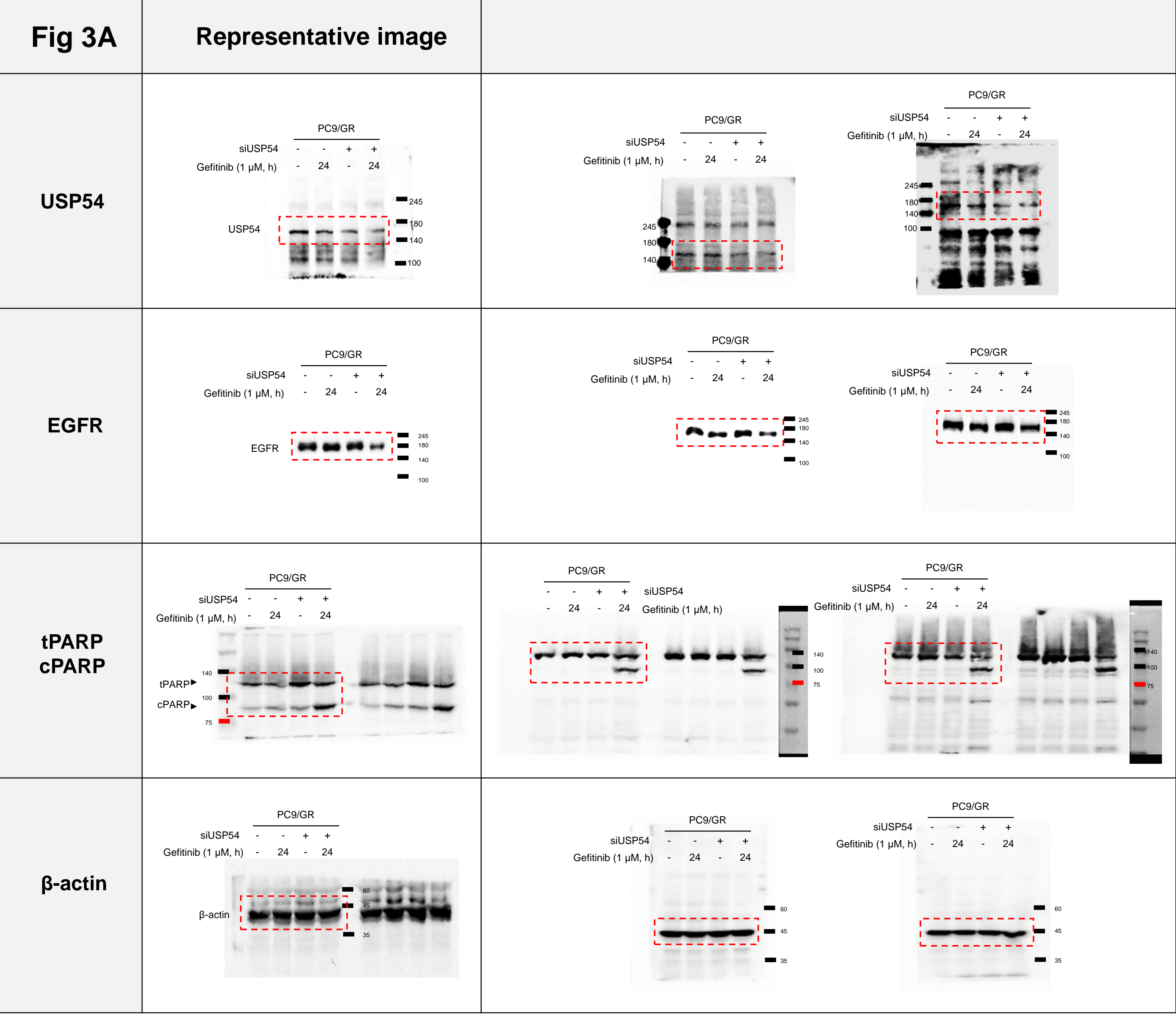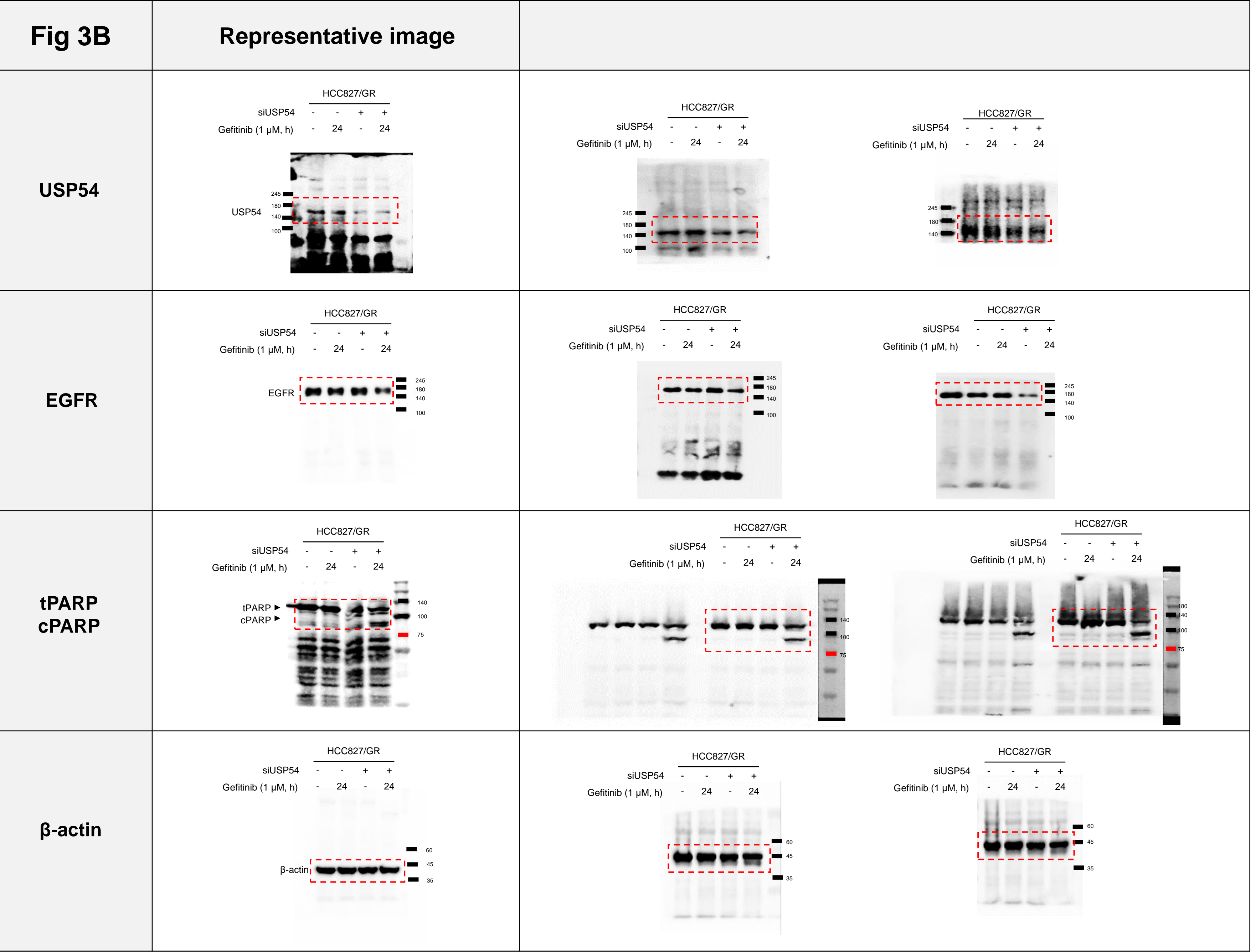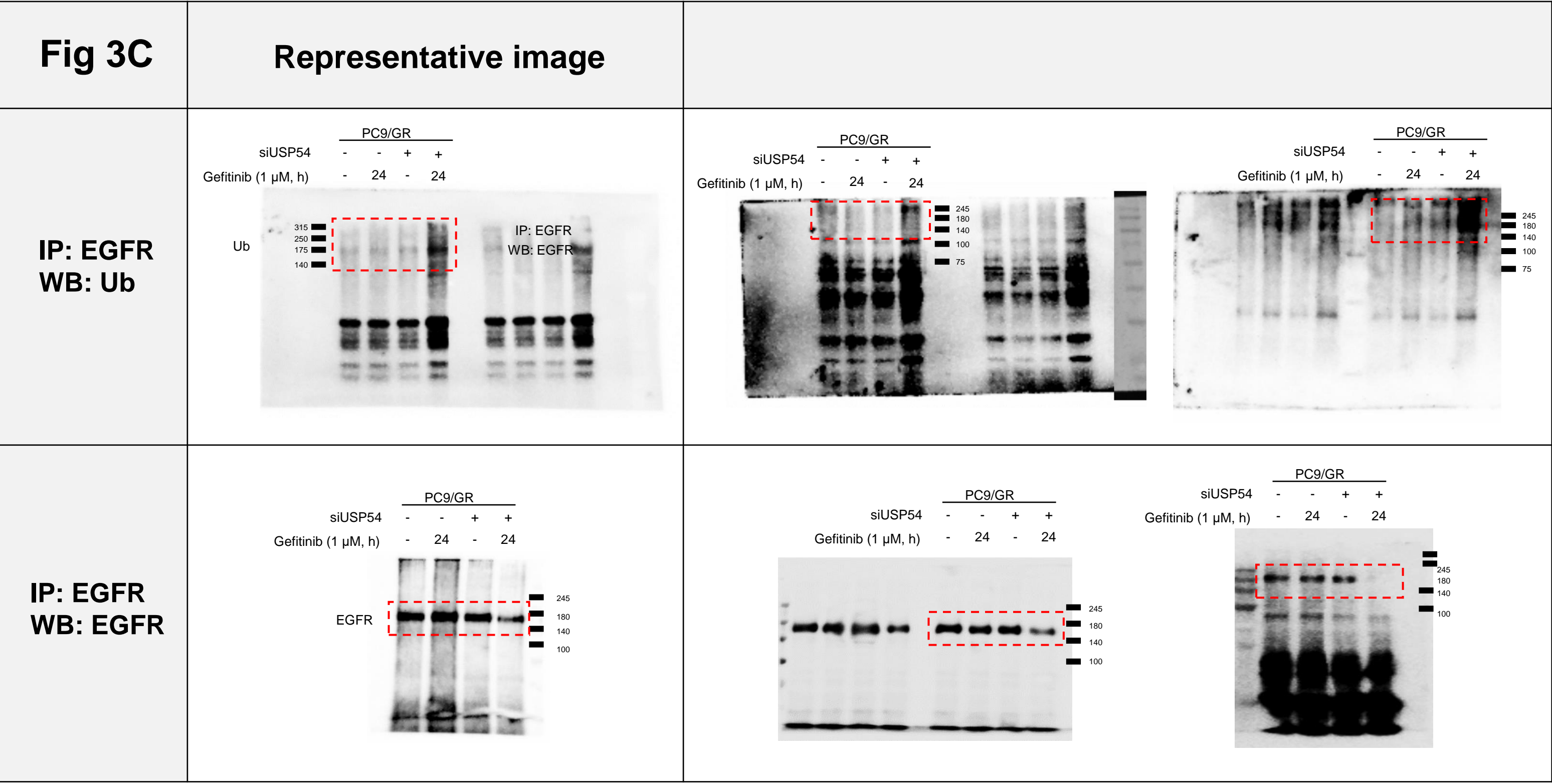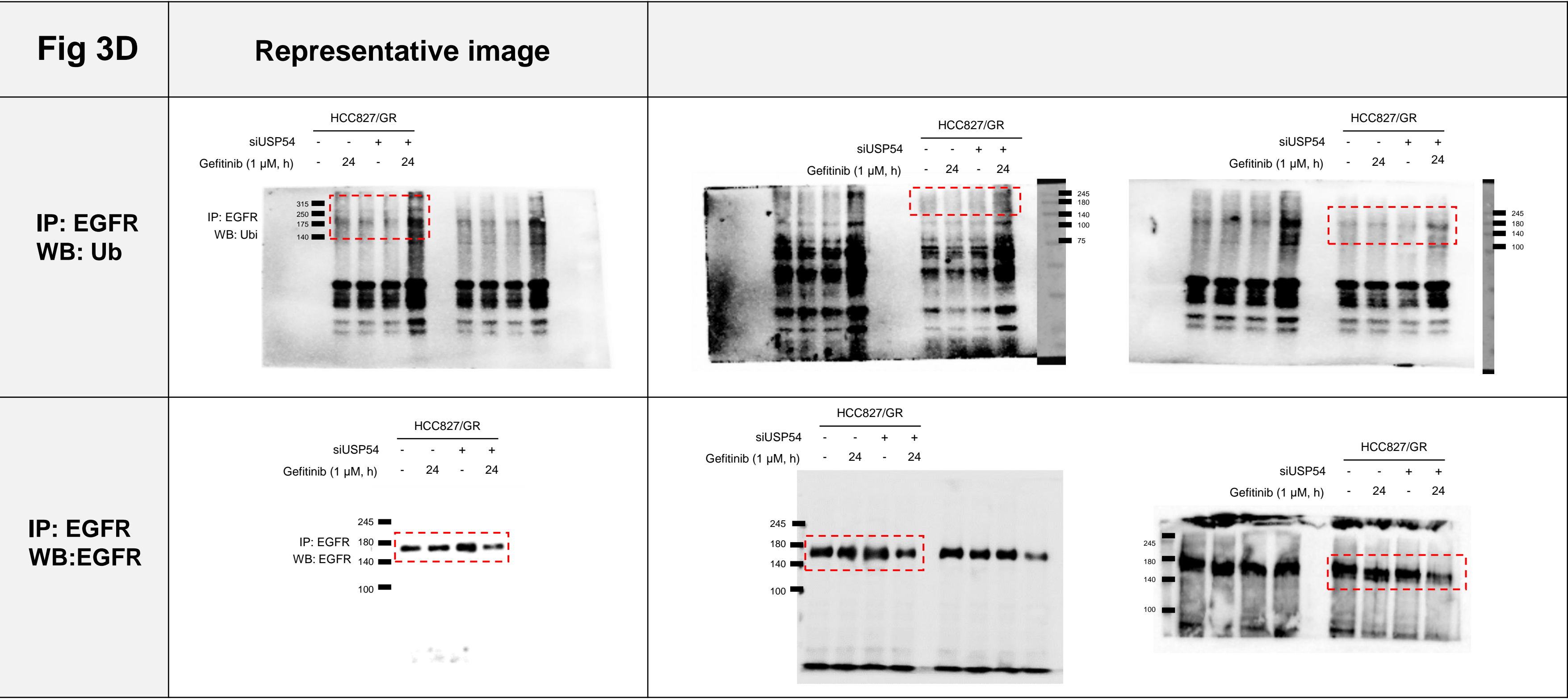

Supplementary figures (Uncropped images)

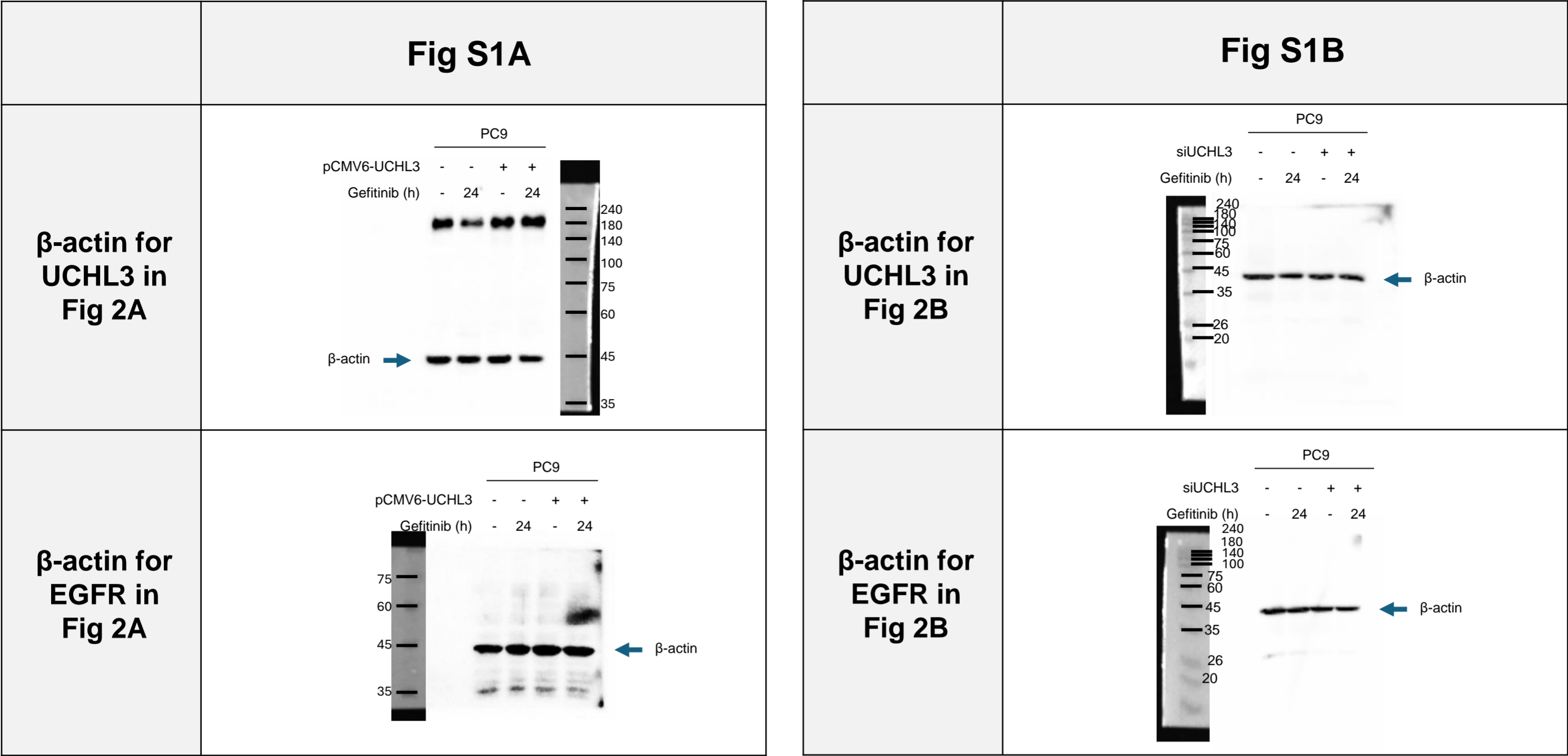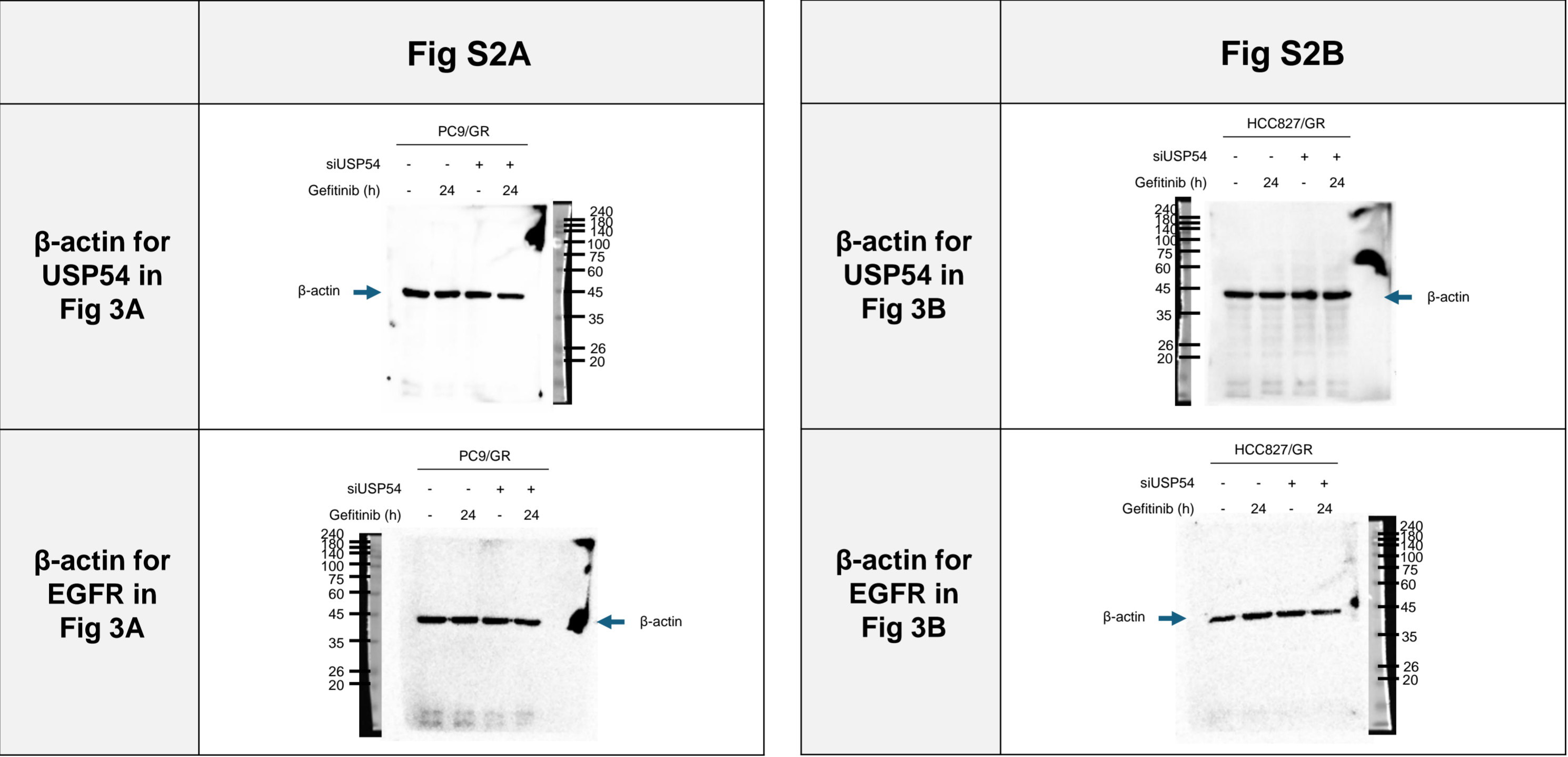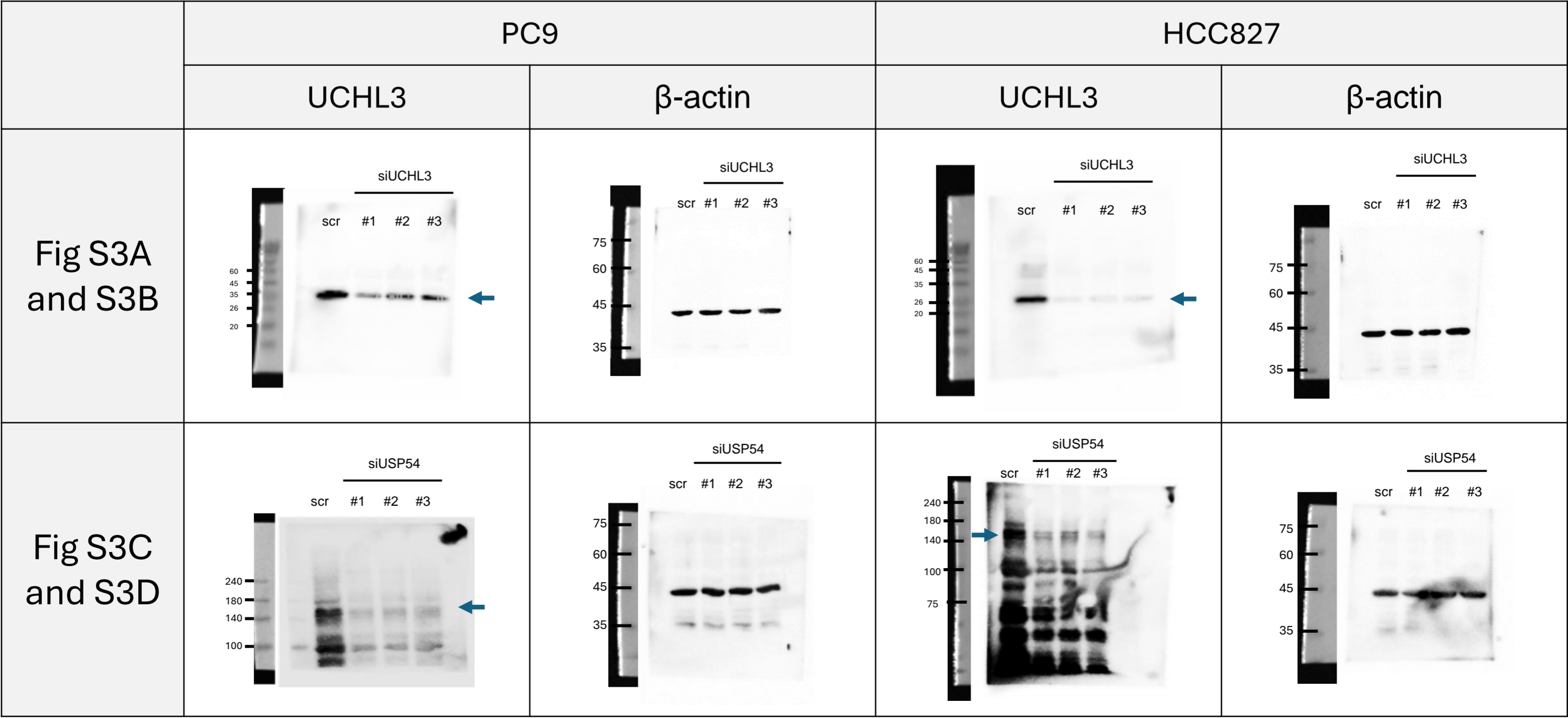

Supplement: S1 Raw Images — (PDF) [file pone.0320668.s006.pdf]
